# Evaluation of the I-PLAN Intervention to Promote Hearing Aid Use in New Adult Users: a Randomized Controlled Trial

**DOI:** 10.1097/AUD.0000000000001195

**Published:** 2022-01-06

**Authors:** Afzarini H. Ismail, Christopher J. Armitage, Kevin J. Munro, Antonia Marsden, Piers D. Dawes

**Affiliations:** 1 Manchester Centre for Audiology and Deafness, School of Health Sciences, University of Manchester, Oxford Road, Manchester, United Kingdom; 2 Department of Audiology and Speech-Language Pathology, Kulliyyah of Allied Health Sciences, International Islamic University Malaysia, Pahang, Malaysia; 3 Manchester University Hospitals NHS Foundation Trust (MFT), Manchester Academic Health Science Centre, Manchester, United Kingdom; 4 Manchester Centre for Health Psychology, School of Health Sciences, University of Manchester, United Kingdom; 5 Centre for Biostatistics, School of Health Sciences, University of Manchester, United Kingdom; 6 NIHR Greater Manchester Patient Safety Translational Research Centre, University of Manchester, Oxford Road, Manchester, United Kingdom.

**Keywords:** Adult patient, Hearing aid use, Randomized controlled trial

## Abstract

**Objective:**

Provision of information is already part of standard care and may not be sufficient to promote hearing aid use. The I-PLAN is a behavior change intervention that is designed to promote hearing aid use in adults. It consists of a prompt, an action plan and provision of information. The objective was to test the effectiveness of the I-PLAN prompt and plan components in promoting hearing aid use and benefit. Hypotheses were: there would be greater hearing aid use, benefit, self-regulation, and hearing aid use habit among participants who received the prompt or plan component, compared with no prompt or no plan component, and the effect would be the greatest in participants who received both prompt and plan; and self-regulation and habit would mediate the effect of prompt and/or plan components on hearing aid use and benefit.

**Design:**

A 2 x 2 factorial randomized controlled trial design. Two hundred forty new adult patients (60 in each group) were randomized to: information (info) only; info + prompt; info + plan; or info + prompt + plan. All participants received treatment as usual in addition to I-PLAN components, which were provided in a sealed envelope at the end of the hearing aid fitting consultation. Participants in the prompt group were instructed to use their hearing aid box as a physical prompt to remind them to use the device. Participants in the plan group were instructed to write an action plan to encourage them to turn their intentions into action. Participants, audiologists, and researchers were blinded to group allocation. The primary outcome was self-reported proportion of time hearing aids were used in situations where they had listening difficulties. Secondary outcomes were hearing aid use derived from data logging, self-reported hearing aid benefit, self-reported self-regulation, and habit. Outcomes were measured at 6-week post-fitting.

**Results:**

Contrary to predictions, participants who received the prompt component reported using their hearing aid *less* than participants without the prompt (*p* = 0.03; *d* = 0.24). The mean proportion of time hearing aid were used was 73.4% of the time in the prompt group compared with 79.9% of the time in the no prompt group. Participants who received the plan component reported using their hearing aids more frequently than those who did not receive the plan (*Mean*_*plan*_ = 81.0% *vs Mean*_*noplan*_ = 71.8% of the time*; p* = 0.01; *d* = 0.34). Receiving both prompt and plan components did not change self-reported proportion of time hearing aids were used but data-logging use was significantly reduced. The prompt reduced self-regulation of hearing aid use compared with the no prompt (*p* = 0.04; *d* = 0.28), while the plan promoted stronger hearing aid use habits than the no plan group (*p* = 0.02; *d* = 0.30).

**Conclusions:**

Audiologists should consider using action plans to promote hearing aid use. Despite the decrease in hearing aid use when using the hearing aid box as a physical prompt, hearing aid use was still high (≈70% of the time). The hearing aid box may have slightly reduced hearing aid use by undermining self-regulation. Participants may have delegated responsibility for hearing aid use to the prompt. Subsequent studies should evaluate different prompts and test the long-term benefit of the plan on hearing aid use via habit formation.

## INTRODUCTION

It is well established that hearing aids are effective in improving hearing-related quality of life (Chisolm et al. 2007; Ferguson et al. 2017). Yet, 5% to 24% of adult hearing aid users do not use their hearing aid (Hartley et al. 2010; Hougaard & Ruf 2011; Aazh et al. 2015; Solheim & Hickson 2017) and up to 40% of adult new hearing aid users use their hearing aids for fewer than 4 hours/day (Aazh et al. 2015). One key reason for non- and under-hearing aid use includes forgetting to use hearing aids (McCormack & Fortnum, 2013). Forgetting to use hearing aids may be a result of patients not making explicit plans for hearing aid use (Sawyer et al. 2019). A Cochrane systematic review of intervention studies to promote hearing aid use found no self-management support and/or service delivery interventions in audiology that were effective in promoting hearing aid use in adults (Barker et al. 2016c). One of the reasons the intervention studies may fail to promote hearing aid use is because of limited use of behavior change theory to inform intervention development (Armitage et al. 2017). It is therefore imperative to develop theory-based interventions to maximize hearing aid use, promote quality of life, and reduce waste of hearing health care resources.

The I-PLAN was the first theory-based intervention designed to promote hearing aid use (Barker et al. 2016a). The I-PLAN intervention was based on the behavior change wheel, a framework for designing and developing a behavior change intervention (Michie et al. 2014). The I-PLAN consists of three main components which are: (1) provision of information related to consequences of hearing aid use and non-use (“info”); (2) provision of a prompt to remind patients to use their hearing aids (“prompt”); and (3) creation of an action plan for hearing aid use (“plan”) (Barker et al. 2016a). The I-PLAN intervention proposed by Barker et al. (2016a) involves audiologists delivering the three components to adult hearing aid patients during hearing aid fitting consultations. The effectiveness of the I-PLAN intervention with audiologists’ involvement has already been tested using a quasi-randomized controlled trial with two arms (Ismail et al. 2021). Participants in the I-PLAN group received all three components of the I-PLAN face-to-face from their audiologist. Ismail et al. (2021) found no significant difference in hearing aid use or benefit among adult patients in the I-PLAN group compared with the Standard Care group. This may have occurred because some components of the I-PLAN might interact with each other to reduce the effectiveness of the intervention (Michie et al. 2018). However, which components of the I-PLAN might reduce the effectiveness of other components is unclear. A study with a factorial design that could separate and examine effects of each component on hearing aid use is needed as recommended by the National Institute for Health and Care Excellence (NICE 2014). Establishing which components are effective in promoting hearing aid use would provide an evidence base and motivate audiologists to include the component(s) of the I-PLAN in clinics as well as maximizing the efficiency of the intervention.

To understand the mechanism or process by which an intervention produces its effects, there is a need to identify the potential mechanisms of action so that interventions may be optimized (Bauman et al. 2002; Michie et al. 2014; Moore et al. 2015). Given the goal of the I-PLAN is to support adult patients in translating their motivation to use hearing aids into actual use, the present study seeks to test a potential mediator that relates to translating motivation into behavior: self-regulation—which includes awareness of standards and self-monitoring; Sniehotta et al. 2006. In addition, given that habits have been shown to mediate the effects of action plans on smoking cessation (Armitage 2016), and habits are likely to be formed when behavior is repeated consistently in a consistent context (Gardner et al. 2012), we aimed to assess hearing aid habit as a second potential mediator.

### Aim and Hypotheses

We aimed to examine the effectiveness of the prompt and plan components of the I-PLAN intervention, measured via self-reported proportion of time hearing aids were used at 6 weeks post-fitting in new adult UK National Health Service (NHS) hearing aid users. Data-logged hearing aid use and hearing aid benefit (measured by self-reported questionnaires) were secondary outcomes. We also aimed to explore whether self-regulation and habit might mediate the effect of the I-PLAN prompt and plan components on hearing aid use and benefit. In this study, we used a 2 x 2 factorial design with prompt and plan as factors. We hypothesized that participants who received:

1. The prompt component would report and show greater improvements in outcome measures (e.g., hearing aid use and benefit and potential mediators; self-regulation and habit) than those with no prompt;
2. The plan component would report and show greater improvements in outcome measures (e.g., hearing aid use and benefit and potential mediators; self-regulation and habit) than those with no plan;
3. Both the plan and prompt would report and show greater improvements in outcome measures (e.g., hearing aid use and benefit and potential mediators; self-regulation and habit) than prompt and plan alone; and
4. Self-regulation and habit might mediate the effect of the I-PLAN intervention on hearing aid use and benefit.

## MATERIALS AND METHODS

### Trial Design

This was a randomized controlled trial with a 2 x 2 factorial design. Participants were randomized to receive one of four possible combinations of the I-PLAN, namely: (1) info only; (2) info + prompt; (3) info + plan; or (4) info + prompt + plan. The first between-subjects factor was *prompt*, which had two levels: (1) prompt, in which participants were from the info + prompt and info + prompt + plan groups; and (2) no prompt, in which participants were from the info only and info + plan groups (Table 1). The second factor was *plan*, which also had two levels: (1) plan, in which participants were from the info + plan and info + prompt + plan groups; and (2) no plan, in which participants were from the info only and info + prompt groups.

**TABLE 1.** The between-subject factors

Groups	No Prompt (n = 120)	Prompt (n = 120)
No plan (n = 120)	(1) Info only (n = 60)	(2) Info + Prompt (n = 60)
Plan (n = 120)	(3) Info + Plan (n = 60)	(4) Info + Prompt + Plan (n = 60)

Each of the four intervention combinations were delivered at the hearing aid fitting appointment. All four groups received treatment as usual in addition to the I-PLAN component. Outcome assessments occurred at 6-week post-intervention. This study was approved by the NRES Committees—West of Scotland (Ref: 17/WS/0253). This study was registered in the clinical trials database (ClinicalTrials.gov Identifier: NCT03742609).

### Setting and Location

The study took place in an audiology department in a single NHS hospital in the north of England, United Kingdom. The study was conducted from February to December 2018.

### Participants

The number of adult patients required for this study was calculated using the G-power software (Faul et al. 2007). The sample size calculation suggested that 180 adult patients (with 45 participants per group) would provide 80% statistical power to detect a significant difference in hearing aid use between the groups (*α* = 0.05) with a medium-sized effect, 0.25, and four covariates (age, sex, audiometric hearing thresholds and self-reported hearing handicap) using ANCOVA. However, to allow for 30% of attrition, we aimed to recruit 60 participants to each group. Therefore, in total, 240 participants were recruited to this study.

New adult hearing aid users were invited to participate in the study. Adult patients who (1) were aged 18 years old or above; (2) never used a hearing aid before; (3) had a good understanding of English; and (4) had sufficient mental capacity to provide informed consent based on the audiologist’s opinion were included. Adult patients who were unable to complete the questionnaires (e.g., due to dementia), based on the audiologist’s opinion, and/or presence of medical contraindications for hearing aids, as described by the British Academy of Audiology (BAA 2007), were excluded. No incentive to participate was offered, and all hearing aids and aftercare appointments were provided free of charge consistent with standard UK NHS practice.

### Outcome Measures

Outcome measures were taken at 6-week post-intervention. The 6-week post-intervention period was chosen because it is standard practice in UK National Health Service audiology clinics for patients to be followed up around 6 weeks after hearing aid fitting. Similar outcome measures were used in a previous study that examined the effectiveness of the I-PLAN delivered by audiologists (Ismail et al. 2021). The outcome measures were:

#### Hearing Aid Use in Situations That Caused Listening Difficulty (Unaided)

We adapted one question from the Glasgow Hearing Aid Benefit Profile (GHABP) (Gatehouse, 1999) related to the proportion of time hearing aids were used in situations that patients experienced hearing difficulty; ‘*In a typical situation where you have hearing difficulty, what proportion of time do you wear your hearing aid?’* The proportion of time hearing aid use in challenging listening situations was measured because: (1) the I-PLAN intervention aimed to promote hearing aid use in the specific situation identified by participants (as in the ‘when’ and ‘where’ plan) and (2) hearing aid use according to the patients’ individual needs based on the identified listening situations reflect the ‘optimal hearing aid use’ (Laplante-lévesque et al. 2013). Answers were provided according to five response options; never/not all (0% of the time), about one-fourth of the time (25% of the time), half of the time (50% of the time), three-fourth of the time (75% of the time), or all the time (100% of the time).

#### Hearing Aid Use

Hearing aid use, defined as hours of use per day, was generated automatically from the data logging feature in the hearing aids. The average hours between right and left hearing aids was used for participants fitted with two hearing aids.

#### International Outcome Inventory for Hearing Aids

The International Outcome Inventory for Hearing Aids (IOI-HA; Cox & Alexander 2002) is a self-report measure of hearing aid use and benefit that is commonly used in audiology studies (Perez & Edmonds 2012). The IOI-HA consists of seven questions indexing aspects of hearing aid outcomes: (1) hearing aid use; (2) hearing aid benefit; (3) residual activity limitations; (4) satisfaction; (5) residual participation restrictions; (6) impact on others; and (7) quality of life. In each question, five response options are provided with a score from 1 to 5 (total score 7-35). Higher scores indicate better outcomes.

#### Hearing Handicap Inventory for the Elderly–Screening Version

The Hearing Handicap Inventory for the Elderly–Screening version (HHIE-S; Ventry & Weinstein 1983) is an index self-perceived hearing handicap due to the hearing loss. The HHIE-S is a 10-item questionnaire that assesses the effect of hearing impairment on social and emotional factors. For example; *‘does a hearing problem cause you to feel embarrassed when meeting new people?’* (emotional factor) and *‘does a hearing problem cause you difficulty when listening to TV or radio?’* (social factor). Questions are scored as yes (4 points), sometimes (2 points) or no (0 points). Higher scores indicate greater perceived hearing handicap.

#### Self-Regulation

Self-regulation for hearing aid use was measured using an adapted version of the self-regulation questionnaire on physical activity by Sniehotta et al. (2006). It measures three components of self-regulation: (1) awareness of standards; (2) self-monitoring; and (3) self-regulatory effort. In total, there are six items (two items for each component), each with a seven-point Likert scale (1 = strongly disagree to 7 = strongly agree). All the items began with a statement ‘*During the last six weeks, I*….and followed by each of the component. For example; ‘*During the last 6 weeks, I often had the intention to use my hearing aid(s) on my mind’* (awareness of standards), ‘*During the last 6 weeks, I consistently checked myself to see if I was using my hearing aid(s) often enough’* (self-monitoring) and ‘*During the last six weeks, I tried hard to use my hearing aid regularly’* (self-regulatory effort). Higher scores indicate greater self-regulation of hearing aid use. The scale showed good internal consistency with a Cronbach alpha of 0.78.

#### Self-Report Behavioral Automaticity Index

The self-report behavioral automaticity index (SRBAI) is four-item measure of habit (Gardner et al. 2012). Habit in relation to hearing aid use was measured using the four items that began with a phrase ‘*Using hearing aid(s) is something…’ ‘I do automatically’, ‘...I do without thinking’, ‘…I do without having to consciously remember to use them’ and ‘…I start doing before I realize I am doing it’*. The four items were answered on a seven-point Likert scale (1 = strongly disagree to 7 = strongly agree). The higher scores indicating stronger habit. The scale showed good internal consistency with a Cronbach alpha of 0.94.

#### Fidelity to the Interventions

All participants in this study were asked ‘have you read the information on the card in the white envelope you were given at the hearing aid fitting appointment?’ in the follow-up questionnaire.

### The I-PLAN Written Materials

In the present study, we developed our own I-PLAN written materials as Barker et al. (2016a) did not specify the exact form of the I-PLAN components. We chose to test I-PLAN prompt and plan components via a set of written materials (Table 2) because provision of the I-PLAN as a set of written materials: (1) standardized the intervention across study audiologists; and (2) made it easier for audiologists to incorporate the I-PLAN into clinical practice (written materials allow for patients to self-complete the intervention, minimizing audiologist time required to deliver the intervention). The written materials were provided by audiologists to new hearing aid users at their hearing aid fitting consultation. The details of the I-PLAN written materials were as follows:

**TABLE 2.** The details of the I-PLAN intervention materials (Barker et al. 2016a), as implemented by Ismail et al. (2021)

Components of the I-PLAN	Behavior Change Technique	Written Materials of I-PLAN
Provision of information related to consequences of hearing aid use and non-use	5.3 Information about social and environmental consequences	Hearing aid use will improve
		Your ability to hear others
		Your social interactions
		The lives of those around you by making it easier for them to communicate with you.
		Not using a hearing aid will
		Reduce your ability to hear your family and friends
		Lead you to withdraw from social activities
		Cause stress and increase burden on those around you by making it harder for them to communicate with you.
Provision of a prompt to remind patients to use their hearing aids	7.1 prompts/cues	*My hearing aid reminder*
		Please use your hearing aid box as a reminder to wear your hearing aid(s). For example, you could put your hearing aid box next to the bathroom mirror last thing at night to remind you to wear your hearing aid(s) in the morning.
Creation of a written plan for hearing aid use	1.4 Action planning	*My hearing aid(s*)
		Please plan where and when to wear your hearing aid(s).
		You can choose any place and time but please write your plan in as much detail as possible. Please write your plan in the space provided.
		*Example*: ‘When I have finished brushing my teeth in the morning, then I will wear my hearing aid(s)’
		*Please write your plan in the space provided in the format in the example*.
		When I ……………., then I will wear my hearing aid(s).
		Use the space below if you want to write more than one plan.

#### Information Related to Consequences of Hearing Aid Use and Non-Use

In terms of behavior change techniques, information about hearing aid use/non-use is delivered as ‘*information about social and environmental consequences’* (BCTTv1 5.3; Michie et al. 2013). For example ‘*hearing aid use will improve your ability to hear others’* (positive consequence) and ‘*not using a hearing aid will reduce your ability to hear your family and friends’* (negative consequence). To identify information related to the social and environmental consequences of hearing aid use, current literature on the impact of untreated hearing loss on significant others (e.g., Scarinci et al. 2008; Vas et al. 2017) was used, based on a literature review by the first author. However, given the long list of consequences identified from the literature, three adult patients with hearing loss aged between 50 and 70 years, two significant others and two audiologists with over 10 years of clinical experience each were consulted in order to identify the three most salient consequences to be included in the I-PLAN and to ensure that the materials could be understood by participants (Table 2).

#### A Prompt to Remind Patients to Use Their Hearing Aids

The second component of the I-PLAN was a ‘*prompt or cue’* (BCTTv1 7.1; Michie et al. 2014). Adult patients were explicitly instructed to use their hearing aid box as a reminder to hearing aid use. Instructions with an example were given; ‘*Please use your hearing aid box as a reminder to wear your hearing aid(s). For example, you could put your hearing aid box next to the bathroom mirror last thing at night to remind you to wear your hearing aid(s) in the morning’*.

#### A Written Action Plan for Hearing Aid Use

The third component of the I-PLAN was ‘*action planning’* (BCTTv1 1.4; Michie et al. 2013). Participants were asked to create at least one written action plan concerning ‘where’ and ‘when’ to use their hearing aid(s). Participants were asked to complete the ‘when-then’ statement provided on the I-PLAN written material. The ‘when-then’ instruction was adapted from Gollwitzer’s (1990) instructions as to how to form implementation intentions, namely, to form ‘if-then’ plans. Such ‘if-then’ plans have been applied across various health behaviors (e.g., to reduce self-harm among patients who have attempted suicide, O’Connor et al. 2017, and to prevent emotional eating, Armitage, 2015). One advantage of the approach is that it can be self-completed and does not require a healthcare professional to be present (Armitage, 2008). The details of the I-PLAN intervention written materials are in Table 2. The I-PLAN written materials were the same as those used in our previous study (Ismail et al. 2021).

### Group Allocation

All four groups received the information about the pros and cons of use and non-use of a hearing aid. The information was provided to all participants based on the assumption that: (1) information alone may not be sufficient to lead adult patients to actual hearing aid use (Kelly & Barker 2016); and (2) patients may have received information about the pros and cons of using a hearing aid at the standard hearing aid fitting appointment (Barker et al. 2016b). All participants were treated similarly across the groups so that the impact of research participation effects (McCambridge et al. 2014) could be minimized.

The first group was the (1) info only group. Adult patients allocated in this group received information alone. In the remaining three groups, adult patients were assigned to receive either: (2) prompt; (3) plan; or (4) prompt and plan, in addition to information.

### Procedure

#### Before Hearing Aid Fitting Appointment

Invitation letters, participant information sheets, consent forms, along with baseline questionnaires were posted to adult patients who were scheduled for initial hearing aid fitting appointments. Adult patients were informed that the aim of the study was to discover which parts of the I-PLAN are most helpful in promoting hearing aid use and benefit for people with hearing problems. Patients were also given a general description of the I-PLAN intervention (e.g., ‘*the I-PLAN includes information about the benefits of hearing aid use and the disadvantages of not using a hearing aid and involves making a short plan about the times and places that you will use your hearing aid*’) in the participant information sheet. A general description of the I-PLAN intervention was provided so that participants were blinded to the specific contents of the I-PLAN components in order to minimize the risk of bias.

All these documents were posted at least 2 days prior to each patient’s appointment. Adult patients who decided to take part in the study were asked to sign the consent form, complete the questionnaire (for baseline characteristics and self-reported hearing handicap, HHIE-S) at home and return the completed documents at the hearing aid fitting appointment.

#### At Hearing Aid Fitting Appointment

Participants who consented to participate in the study had their National Health Service (NHS) behind-the-ear hearing aid(s) fitted by their audiologist and received treatment as usual provided by audiologists. Treatment as usual included: (1) programming the hearing aid(s); (2) performing real ear measurements to fine-tune the hearing aid(s); (3) advising patients on realistic expectations (e.g., hearing aid does not restore normal hearing), communication strategies with hearing aid and acclimatization to a hearing aid; (4) demonstrating and providing instruction on hearing aid management (e.g., to on and off the hearing aid); (5) explaining post-fit services (e.g., battery and repair services); (6) providing patients with spare batteries, a hearing aid box and written information about specific hearing aid fitting; (7) providing a brief explanation about follow-up appointments (e.g., discussion of issues related to hearing aid use and management); and (8) scheduling a face-to-face follow-up appointment in 6 weeks after fitting.

The participants were randomly allocated to receive one of the four possible combinations of the I-PLAN materials. The four possible combinations of the I-PLAN materials were sealed in numbered opaque white envelopes. The numbered opaque white envelopes then were randomized by one of the authors using a random number generator (https://www.random.org/integers/). The random number generator was used to determine the order in which the white envelopes were piled. The envelopes were placed in a paper tray at the clinic. Once participants had consented to take part in the study, audiologists took one of the envelopes containing one of the four sets of I-PLAN materials for the participant. The I-PLAN materials were given to the participants at the end of their consultation. In order to minimize any bias, audiologists did not deliver the I-PLAN intervention. Participants were asked to review and/or complete the I-PLAN material(s) by themselves in the consultation room prior to leaving the clinic. Once completed, participants were asked to take the I-PLAN materials with them. All participants were scheduled for a routine face-to-face 6-week hearing aid follow-up appointment. Clinical reception staff managed scheduling of patients for hearing aid fitting and follow-up appointments. In summary, the individual components of the I-PLAN were not discussed with the participants and treatment allocation was concealed from the audiologists and researchers.

#### Post-Hearing Aid Fitting Appointment

Participants were posted a questionnaire prior to their 6-week hearing aid follow-up. The questionnaire included all five self-reported outcome measures (e.g., hearing aid use in difficult situations, IOI-HA, HHIE-S, self-regulation, and habit). Participants were asked to complete the questionnaire and return the completed questionnaire at their follow up appointment to the first author in a sealed envelope. Data on hearing aid usage were downloaded from patients’ hearing aids by their audiologist at the follow-up appointment. Therefore, outcome data were extracted with the research team being blind to group allocation. If participants did not attend their hearing aid follow-up appointment, the follow-up questionnaire was posted again to them. Participants were asked to return the questionnaire directly to the first author.

### Statistical Analysis

Descriptive statistics were reported to summarize the baseline clinical and demographic characteristics of participants. No formal statistical analysis comparing the baseline clinical and demographic characteristics of participants was performed as recommended by CONSORT guidelines (Schulz et al. 2010).

Two analyses were conducted to examine the effectiveness of I-PLAN prompt and plan components on the outcome measures. Initially, a descriptive analysis by randomized group (info only, info + prompt, info + plan and info + prompt + plan) was performed to investigate the possibility of interaction effects of the prompt and plan on each outcome measured across groups. Then, 2 x 2 ANCOVAs were conducted to examine the interaction and main effects of the I-PLAN prompt and plan components on each outcome measure while controlling for clinical and demographic characteristics of participants that may impact outcomes (e.g., age, sex, audiometric hearing thresholds and self-reported hearing handicap, HHIE-S (unaided). Analyses controlled for the baseline clinical and demographic characteristics of participants to increase precision of estimated intervention effects (Lingsma et al. 2010).

We investigated whether the effect of the plan or prompt on hearing aid use (proportion of time or data logging) and hearing aid benefit (IOI-HA or HHIE-S (aided) may be mediated through self-regulation or habit. Where there was evidence of an effect of the prompt/plan on both a potential mediator (self-regulation or habit) and hearing aid outcomes, direct effects and indirect effects of the plan/prompt on the relevant outcome were estimated using the bootstrapping method (Preacher & Hayes 2008) in PROCESS macro version 3.3 for SPSS (Hayes 2012). The bootstrapping method is a procedure that involves multiple resampling of the data to estimate indirect effect and confidence interval of the indirect effect (Preacher & Hayes 2008). The 95% of the confidence interval of the indirect effects was obtained with 5000 bootstrap samples (Preacher & Hayes 2008).

## RESULTS

### Recruitment

**Fig. 1.**
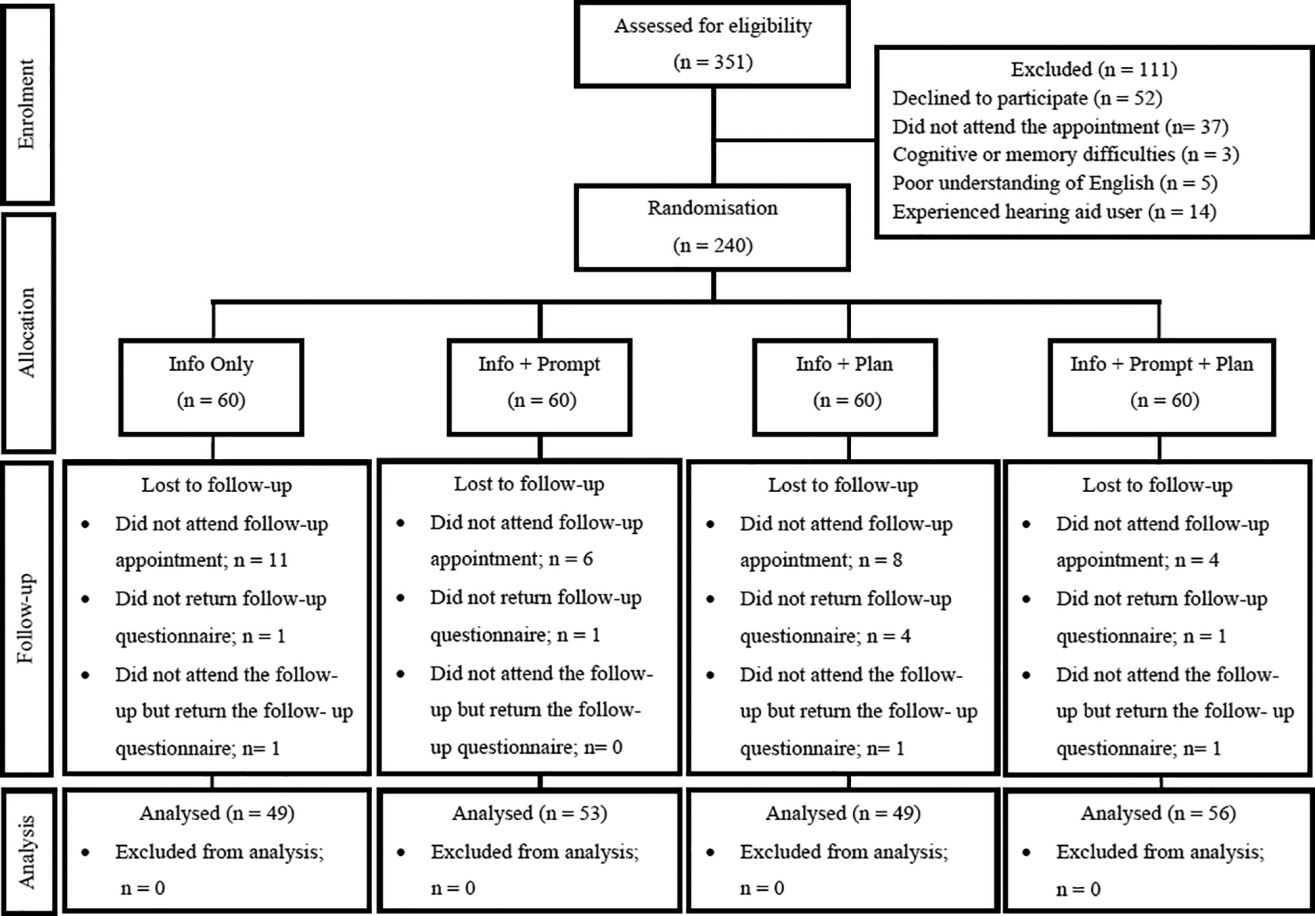
CONSORT diagram showing flow of participants through the trial for main outcome measure, the self-reported hearing aid use (n = 207).

Figure 1 presents the flow of study participants. Of the 351 adult patients who were scheduled for a hearing aid fitting appointment, 52 (14.8%) declined to participate. A further 59 (16.8%) were excluded (did not attend the hearing aid fitting appointment (n = 37), cognitive or memory difficulties (n = 3), poor understanding of English (n = 5) or previous experience with a hearing aid (n = 14)).

In total, 240 (68.4%) first-time hearing aid adult patients were consented to participate in this study. They were randomly allocated to one of the four groups: (1) info (n = 60); (2) info + prompt (n = 60); (3) info + plan (n = 60); and (4) info + prompt + plan (n = 60).

### Clinical and Demographic Characteristics of Participants

Table 3 shows the clinical and demographic characteristics of the patients allocated randomly to the four groups at the hearing aid fitting appointment. All the participants across the groups were first-time hearing aid users and were fitted with the NHS behind-the-ear hearing aid(s). The mean age of participants across the groups was 67.6 years (SD = 13. 35; Range = 22 to 94 years). On average, participants across the groups showed a mild to moderate hearing handicap based on the mean score of the HHIE-S (unaided) questionnaire; 21.7 (SD = 9.52).

**TABLE 3.** Baseline characteristics of the participants at the hearing aid fitting appointment (n = 240)

Variables	Info (n = 60)		Info + Prompt (n = 60)		Info + Plan (n = 60)		Info + Prompt + Plan (n = 60)	
	Mean	SD	Mean	SD	Mean	SD	Mean	SD
Age	67.2	12.02	67.8	13.64	68.4	13.96	66.9	13.96
Age range	26–92		30–88		25–94		22–91	
Pure tone average	35.0	13.02	33.0	12.40	36.5	12.11	31.1	8.39
HHIE-S (unaided)	20.9	9.48	21.8	9.23	21.7	9.58	22.4	9.95
Sex	n	%	n	%	n	%	n	%
Male	32	53.3	28	46.7	25	41.7	26	43.3
Female	28	46.7	32	53.3	35	58.3	34	56.7

Pure tone average = average of hearing thresholds at 0.5, 1, 2, and 4 kHz in the better ear.

HHIE-S, Hearing Handicap Inventory for the Elderly and for Adults – Screening version (Ventry & Weinstein 1983).

Based on the descriptive analysis, the mean age and the mean HHIE-S of participants were similar across groups. The mean pure tone average was slightly higher in the info + plan group and lower in the info + plan + prompt group. The proportion of males was slightly higher in the info only group compared with the other groups.

### Attrition Analyses and Missing Values

In total, 29 out of 240 participants (12.1%) failed to attend their 6-week follow-up appointment. The follow-up data were missing completely at random based on Little’s Missing Completely at Random Test [*X^2^* (6) = 4.36, *p* = 0.63].

Of 211 participants who attended the 6-week follow-up appointment, seven participants (3.3%) did not return the follow-up questionnaire (Fig. 1). Three additional questionnaires were received from three out of 29 (10.3%) participants who did not attend the follow-up. In total 207 follow-up questionnaires were obtained from participants across the four groups.

### Compliance With Interventions

All participants in this study reported that they had read the I-PLAN cards based on the question asked, *‘Have you read the information on the card in the white envelope you were given at the hearing aid fitting appointment?’* in the follow-up questionnaire.

### Effects of the I-PLAN Prompt and Plan Components

The means and standard deviations of outcome measures by randomized group are shown in Table 4. Table 5 summarizes the means and standard deviations of outcome measures as well as adjusted mean and standard errors for each between-subjects factor.

**TABLE 4.** The outcome measures at the 6-week post-intervention across the four randomization groups

Variables	Info only (n = 49)		Info + Prompt (n = 53)		Info + Plan (n = 49)		Info + Prompt + Plan (n = 56)	
	Mean	SD	Mean	SD	Mean	SD	Mean	SD
Proportion of time (%)	73.0	26.44	70.8	31.67	86.7	18.47	75.9	27.37
IOI-HA	27.4	4.24	26.9	5.16	28.8	4.46	27.1	5.39
HHIE-S (aided)	14.3	9.64	15.9	10.52	12.9	9.75	15.9	9.18
Self-regulation	5.0	0.98	4.7	1.22	5.2	1.04	4.9	1.15
SRBAI	4.1	1.77	4.1	1.63	4.9	1.53	4.3	1.78
	(n = 49)		(n = 54)		(n = 52)		(n = 56)	
Data logging (hrs/day)	7.0	4.75	8.5	5.66	8.5	5.20	6.9	4.92

The reported values are “raw” and not adjusted for baseline characteristics.

HHIE-S, Hearing Handicap Inventory for the Elderly and for Adults – Screening version; IOI- HA, International outcome inventory for hearing aids; SRBAI, Self-Report Behavioral Automaticity Index

**TABLE 5.** Main effects of the I-PLAN plan and prompt components on hearing aid outcomes, self-regulation and habit formation at six-week outcome assessment

Variables	Prompt (n = 109)	No Prompt (n = 98)	*p* Value	Plan (n = 105)	No Plan (n = 102)	*p* Value
Proportion of time (%)						
Mean (SD)	73.4 (29.52)	79.9 (23.72)	0.03*	81.0 (24.15)	71.8 (29.15)	0.01*
Mean_adj (_SE)	72.8 (2.43)	80.5 (2.57)		81.4 (2.47)	71.8 (2.51)	
IOI-HA						
Mean (SD)	27.0 (5.25)	28.1 (4.38)	0.13	27.9 (5.03)	27.2 (4.72)	0.22
Mean_adj (_SE)	27.0 (0.46)	28.0 (0.49)		27.9 (0.47)	27.1 (0.47)	
HHIE-S (aided)						
Mean (SD)	15.9 (9.81)	13.6 (9.67)	0.06	14.5 (9.52)	15.1 (10.09)	0.57
Mean_adj (_SE)	15.8 (0.80)	13.7 (0.85)		14.4 (0.82)	15.1 (0.83)	
Self-regulation						
Mean (SD)	4.8 (1.18)	5.1 (1.01)	0.04*	5.0 (1.10)	4.9 (1.12)	0.35
Mean_adj (_SE)	4.8 (0.10)	5.1 (0.11)		5.0 (0.10)	4.9 (0.11)	
SRBAI						
Mean (SD)	4.2 (1.70)	4.5 (1.70)	0.29	4.6 (1.69)	4.1(1.69)	0.02*
Mean_adj (_SE)	4.2 (0.16)	4.5 (0.16)		4.6 (0.16)	4.1 (0.16)	
	(n = 110)	(n = 101)		(n = 108)	(n = 103)	
Data-logging (hours/day)						
Mean (SD)	7.7 (5.33)	7.8 (5.03)	0.91	7.7 (5.10)	7.8 (5.28)	0.97
Mean_adj (_SE)	7.7 (0.48)	7.8 (0.50)		7.8 (0.48)	7.7 (0.49)	

The mean (SD) values are derived from the descriptive table and are not adjusted for covariates. Mean_adj_ (SE) values are derived from estimated marginal means tables and adjusted for age, sex, audiometric hearing thresholds and self-reported hearing handicap, HHIE-S (unaided).

*p < 0.05.

HHIE-S, Hearing Handicap Inventory for the Elderly and for Adults – Screening version; IOI- HA, International outcome inventory for hearing aids; SRBAI, Self-Report Behavioral Automaticity Index.

The effects of the I-PLAN prompt and plan components on outcome measures as follows:

#### Hearing Aid Use in Situations That Caused Listening Difficulty (Unaided)

Of 207 participants who completed their follow-up questionnaire, the mean proportion of time hearing aids were used in situations that caused listening difficulty was 76.5% of the time (SD = 27.06). Of the 207 participants, 46.9% (97 out of 207) reported that they used their hearing aids in situations that caused listening difficulty for ‘all the time’ since hearing aids were fitted, 24.6% for ‘three-fourth of the time’, 17.9% for ‘half of the time’, 8.7% for ‘one-fourth of the time’, and only 1.9% ‘never/ not at all’ used their hearing aids. Of those who reported never having used their hearing aids, three of the participants were in the info + prompt group (n = 3) and one in the info group (n = 1).

The mean proportion of time hearing aids were used was higher in the info + plan group (Mean_plan_ = 86.7% of the time, SD = 18.47) and info + prompt + plan group (Mean_info+prompt+plan_ = 75.9% of the time, SD = 27.37) compared with the info only group (Mean_info_ = 73.0% of the time, SD = 26.44). The mean proportion of time hearing aids were used was slightly lower in the info + prompt only group (Mean_prompt_ = 70.8% of the time, SD = 31.67) than the info only group.

Based on 2 X 2 ANCOVA, there was no statistically significant interaction effect of the prompt and plan on proportion of time hearing aids were used. However, there was a main effect of the prompt on the proportion of time hearing aids were used [*F* (1, 199) = 4.65; *p* = 0.03; *Partial η^2^* = 0.02, *d* = 0.24]. Participants in prompt group reported a lower proportion of time hearing aids were used. There was also a significant main effect of the plan on the proportion of time of hearing aids were used [*F* (1, 199) = 7.40; *p* = 0.01; *Partial η^2^* = 0.04, *d* = 0.34]. Participants in the plan group reported a higher proportion of time hearing aids were used (Table 5).

#### Data-Logged Hearing Aid Use

For data logging, the mean hearing aid use averaged over 6 weeks across participants who attended the follow up appointment (n = 211) was 7.7 (SD = 5.17) hours/day. The mean hearing aid use was higher in the info + plan (Mean_plan_ = 8.5 hours/day, SD = 5.20) and info + prompt (Mean_prompt_ = 8.5 hours/day, SD = 5.66) groups compared with the info only group (Mean_info_ = 7.0 hours/day, SD = 4.75). However, the mean data-logged hearing aid use for the info + plan + prompt (Mean_info+prompt+plan_ = 6.9 hours/day, SD = 4.92) was similar to the info only group, suggesting an interaction between plan and prompt on data-logged hearing aid use. In addition, the hearing aid use among seven participants who did not return the follow-up questionnaire was 6.7 (SD = 5.29) hours/day.

Based on the data-logging, 154 of 211 participants (73.0%) used their hearing aid more than 4 hours/day and were considered as ‘regular hearing aid users’ (Aazh et al. 2015). Based on MANOVA [for age, pure tone average and HHIE-S (unaided)], there was a significant difference between regular and non-regular hearing aid users [*F* (3, 207) = 3.05; *p* = 0.03; *Pillai’s trace V* = 0.04; *Partial η^2^* = 0.04]. Participants who used their hearing aid(s) regularly reported higher self-perceived hearing handicap score than participants who used their hearing aid(s) non-regularly [HHIE-S score (unaided); regular hearing aid users, Mean_regular_ = 22.5, SD = 9.38, non-regular hearing aid users, Mean_non-regular_ = 18.4, SD = 9.36; *p* = 0.01] (Supplemental Digital Content 1, http://links.lww.com/EANDH/A996).

There was a statistically significant interaction between prompt and plan components of the I-PLAN on hearing aid use measured via data-logging [*F* (1, 203) = 5.13; *p* = 0.03; *Partial η^2^* = 0.03] while controlling for clinical and demographic characteristics of participants. Participants who received both the prompt and plan components of the I-PLAN had reduced hearing aid use measured via data-logging compared with participants who received the prompt or plan only (Table 4). There was no significant main effect of the prompt or plan on data-logged hearing aid use.

#### Hearing Aid Benefit Based on IOI-HA and HHIE-S (Aided)

The mean hearing aid benefit based on IOI-HA and HHIE-S (aided) were similar across all randomization groups. However, in the info + plan group, mean HHIE-S (aided) was slightly lower compared with other groups. There were no statistically significant interaction effects of the prompt and plan components on hearing aid benefit; IOI-HA [*F* (1, 199) = 1.10; *p* = 0.30; *Partial η^2^* = 0.01] or HHIE-S (aided) [*F* (1, 199) = 0.21; *p* = 0.65; *Partial η^2^* = 0.00]. There were also no significant main effects of the prompt or plan on hearing aid benefit (Table 5).

#### Potential Mechanism of Action; Self-Regulation and Habit

The mean self-regulation and habit of hearing aid use (based on SRBAI) were similar across the four randomization groups. However, the mean habit was slightly higher in the info + plan group compared with other groups. There were no statistically significant interaction effects of the prompt and plan components on self-reported self-regulation [*F* (1, 199) = 0.01; *p* = 0.92; *Partial η^2^* = 0.00] and habit [*F* (1, 199) = 2.20; *p* = 0.14; *Partial η^2^* = 0.01], after controlling for clinical and demographic characteristics of participants. Analysis with 2 X 2 ANCOVA however, showed there was a main effect of the prompt on self-regulation score [*F* (1, 199) = 4.40; *p* = 0.04; *Partial η^2^* = 0.02, *d* = 0.28]. Participants in prompt group reported lower self-regulation hearing aid use compared with the participants in no prompt group (Table 5). There was also a significant main effect of the plan on habit of hearing aid [*F* (1, 199) = 6.07; *p* = 0.02; *Partial η^2^* = 0.03, *d* = 0.30]. Participants in the plan group reported higher habit scores compared with no plan group (Table 5).

### Potential-Mediating Effects

Given there were statistically significant effects of: (1) prompt on the proportion of time hearing aids were used and self-regulation score; and (2) plan on the proportion of time of hearing aids were used and habit score, mediation analyses were conducted. The aims were to test whether: (1) self-regulation may mediate the effect of prompt on the proportion of time hearing aids were used; and (2) habit may mediate the effect of plan on the proportion of time hearing aids were used.

The independent variables were groups; prompt (dummy-coded as 0 = no prompt, 1 = prompt) and plan (dummy-coded as 0 = no plan, 1 = plan). The mediators were self-regulation and habit (SRBAI) of hearing aid use. The dependent variable was proportion of time hearing aids were used. The covariates, age, sex, audiometric hearing thresholds and self-reported hearing handicap, HHIE-S (unaided), were included in the analysis.

The results showed the confidence intervals associated with the indirect effects of self-regulation and habit did not contain zero. The results suggested that: (1) self-regulation mediated the effect of prompt on the proportion of time hearing aids were used (95% CI = −5.46 to −0.11) and (2) habit mediated the effect of plan on the proportion of time hearing aids were used (95% CI = 0.78 to 7.67). The results therefore indicated that: (1) the prompt undermined self-regulation of hearing aid use and (2) the plan promoted hearing aid use habits.

## DISCUSSION

This is the first randomized controlled trial investigating the efficacy of the prompt and plan components of the I-PLAN health behavior change intervention to promote hearing aid use and benefit in new adult patients fitted with hearing aids. There are five key findings:

1. The proportion of time hearing aids were used in specific situations that caused listening difficulty was *lower* in participants who received the prompt compared with no prompt [Mean_prompt_ = 73.4% vs Mean_noprompt_ = 79.9% of the time]
2. Participants who received the plan reported greater hearing aid use compared with no plan [Mean_plan_ = 81.0% vs Mean_noplan_ = 71.8% of the time];
3. Data-logged hearing aid use was reduced when participants received both prompt and plan;
4. The prompt reduced self-regulation of hearing aid use; and
5. The plan promoted hearing aid use habits.

In general, all participants involved in this study, regardless of their intervention group, showed relatively high hearing aid use (≈70% of the time). Contrary to predictions, the proportion of time hearing aids were used was *lower* in the prompt group compared with the no prompt group (73.4% versus 79.9% of the time; *d* = 0.24). Those who received the prompt also reported lower self-regulation scores. Results suggest that the prompt adversely affected hearing aid use by undermining self-regulation. It is possible that the physical prompt caused participants to delegate the responsibility for hearing aid use to the prompt rather than having to intrinsically monitor, evaluate and adjust their hearing aid use. Although consistent with relevant theory (e.g., Powers 1973), because self-regulation and hearing aid use were measured at the same point, we cannot be certain that the reduced self-regulation impacted hearing aid use. Longitudinal data at more time points would be needed to investigate whether reduced self-regulation preceded reduced hearing aid use (Lee et al. 2019, Sniehotta et al. 2006). In addition, given stigma associated with age may adversely impact hearing aid use (Wallhagen 2010), perhaps the physical prompt chosen in this study (i.e., the hearing aid box) was perceived by participants as an aversive reminder of aging. Participants in the prompt group may have used their hearing aid less as a strategy to avoid a psychological threat associated with aging. Those who received the physical prompt therefore were less likely using their hearing aid and to self-regulate of their hearing aid use. The unexpected adverse negative impact of the physical prompt suggests caution is warranted before delivering physical prompt behavior change interventions in hearing as well as in relation to other health behaviors. The negative impact of the specific physical prompt chosen in this study suggests that not just any object could serve as a physical prompt. Rather, researchers should identify physical prompts that are relevant to participants and do not have any negative associations to ensure that the physical prompt does not have adverse effects on desired health behavior.

Consistent with our hypothesis, participants who wrote hearing aid use plans reported a greater proportion of time hearing aids were used in situations that caused listening difficulty compared with participants who did not receive the plan component (80.95% versus 71.81%; *d* = 0.34), at 6 weeks post-fitting. The positive impact of the plan on hearing aid use is in line with research outside audiology on the impact of plan interventions on health-behavior change [for example; physical activity (Bélanger-Gravel et al. 2013), smoking cessation (Armitage & Arden 2008), and alcohol consumption (Armitage & Arden 2012)]. Based on the mediation analysis, the effect of the plan component on hearing aid use may be attributable to hearing aid use habit. Previous research similarly showed that habit mediated the effects of a behavioral plan on smoking cessation (Armitage 2016). Writing and using a specific plan for when and where to use hearing aids may provide a positive context for participants to use their hearing aid and hence facilitate hearing aid use habits, which were observable at 6 weeks (42 days) post fitting as in the present study. Humes et al. (2009) suggested self-reported measures stabilize 3 to 6 months post-fitting. Note that previous research outside audiology indicated that habits take on average 66 days to form (Lally et al. 2010). The time course of habit formation in relation to hearing aids may warrant further investigation; understanding of the time course and dynamics of habit formation in relation to hearing aid use may help identify opportunities for intervention to facilitate habit and promote hearing aid use.

The effect size of the plan component was ‘small to medium’ (*d* = 0.34) according to Cohen’s (1992) criteria. Whether the result represents a clinically significant change is unclear. Future research would need to establish a minimal clinically important difference in the proportion of time hearing aids are used in challenging listening environments that is relevant to hearing aid users (Armijo-Olivo 2018). The small to medium effect size found in this study may also indicate the complex nature of hearing aid use and the limits of a single behavior change strategy in promoting hearing aid use. Participants may experience a variety of difficulties that could impact hearing aid use (e.g., comfort, changing batteries; McCormack & Fortnum 2013). Interventions to promote hearing aid use likely need to include plan behavior change elements along with strategies to identify and manage other psychological and practical factors that may limit hearing aid use (e.g., the COM-B model; Michie et al. 2014). One might also increase the effectiveness of the plan component by asking participants to create a behavioral plan that contains more than two elements (e.g., when and where). A previous review study found plans that contain four or five components (e.g., with whom, frequency, intensity and duration) may be more effective in promoting health behaviors than plans containing two components (Carraro & Gaudreau 2013).

We found that the behavior change techniques (‘*action planning’* (BCTTv1 1.4) and ‘*prompt’* (BCTTv1 7.1)) interacted with each other in reducing the effectiveness of the I-PLAN intervention in promoting hearing aid use measured via data-logging. The result is in line with Ismail et al.’s (2021) finding that providing all the I-PLAN components together did not result in greater hearing aid use or benefit than standard care. Given that prompt reduced hearing aid use and plan increased hearing aid use, it is possible that the effect of making specific plans was undermined by providing prompts. This possibility could be explored by asking participants to describe their experience of the intervention in detail.

### Strength and Limitations of the Study

The first strength of this study is that the participants were randomized to the intervention. A second strength is that I-PLAN components were not shared with the participants or audiologists and treatment allocation was concealed from the researchers and audiologists. This study also had sufficient sample size to detect small to medium effects of the I-PLAN intervention components on hearing aid use, and the age of the sample was representative of the majority of first-time hearing aid users in the UK (Action on Hearing Loss 2018).

Potential limitations were first, the plan created by adult patients or how participants used their hearing aid box as a prompt was not examined at the 6-week follow-up. Therefore, we cannot ascertain whether participants had carried out their plans or how they used their hearing aid box as a prompt (e.g., whether the hearing aid box stayed in the one place or was moved around). However, given the results of the present study revealed that there was a significant difference between participants in the plan group compared with no plan group on the proportion of time of hearing aids were used in challenging listening situations, it seems reasonable that participants in the plan group used their hearing aids as they had planned. Qualitative interviews with participants may provide understanding of the plans created and used by participants to promote hearing aid use. Qualitative interview with participants who received the prompt component also may give us insight about how participants had perceived their hearing aid box (e.g., positive prompt or negative prompt) or used their hearing aid box. Second, short-term (i.e., 6 weeks) outcomes were measured. It would be valuable to examine whether the effect of the plan persists in the long-term (e.g., 3, 6, and/or 12 months). Third, due to time constraints, an additional standard care group (receive none of the I-PLAN component) was not included as it would have added to the number of participants needed and have extended the period of data collection beyond feasible time limits. As a result, we were unable to quantify whether the outcomes for each I-PLAN group were different to those for standard care. Future research should consider including a standard care control group. In addition, future research could also examine the separate effects of the plan and prompt components of the I-PLAN when delivered by audiologists compared with patients completing the I-PLAN on their own. Fourth, as the mediator and the outcomes were measured at the same point, we cannot be certain that the meditator causes the outcome. Data at more time points would be needed to investigate this further (Lee et al. 2019).

## CONCLUSION

This is the first randomized controlled trial of an intervention to promote hearing aid use based on a theoretical model of behavior change. Provision of a hearing aid box as a physical prompt reduced hearing aid use and self-regulation. Future research is required to understand the negative impact of physical prompts on hearing aid use and self-regulation by randomizing participants to different kinds of prompts. Planning, a volitional strategy, did promote hearing aid use in listening situations that users find most challenging and promote hearing aid use habit. Audiologists should identify other volitional behavior change strategies that might promote hearing aid outcomes. Behavior change strategies that promote patient hearing aid outcomes could be included in audiological education and training programs and provide an evidence base for clinical guidelines.

## ACKNOWLEDGMENTS

We thank the audiology team at Manchester University Hospitals NHS Foundation Trust for their help in recruiting participants. The authors also thank Prof. Harvey Dillon and Dr Fiona Baker for their support and advice.

## Supplementary Material


