# Supplementary material for: Evaluation of the I-PLAN Intervention to Promote Hearing Aid Use in New Adult Users: a Randomized Controlled Trial
Source: Ear Hear. 2022 Jan 6;43(4):1103–13. doi: 10.1097/AUD.0000000000001195 (PMC9197143; doi:10.1097/AUD.0000000000001195)
Supplement: Supplementary file 1 [file aud-43-1103-s001.pdf]

Supplemental Digital Content 1: Table that shows clinical and demographic characteristics of regular and non-regular hearing aid (HA) users.

**Variables**	**Regular HA users (n = 154)**		**Non-regular HA users (n= 57)**		**Statistical value**	
	Mean	SD	Mean	SD	*F*	p^a
Age	68.6	13.57	67.5	11.53	0.31	0.58
Pure tone Average*	33.7	11.76	32.7	11.88	0.41	0.52
HHIE-S (unaided)	22.5	9.38	18.4	9.36	8.23	0.01**
Gender	n	%	n	%	X^2	p^a
Male	76	49.40	24	42.10	0.88	0.35
Female	78	50.60	33	57.90		

*Pure tone average = Average of hearing thresholds at 0.5, 1, 2 and 4 kHz in the better ear. HHIE-S = Hearing Handicap Inventory for the Elderly and for Adults – Screening version (Ventry & Weinstein 1983). a = p-values are from the univariate ANOVAs (F) or chi-square test (X^2). All comparisons are non-significant except for the HHIE-S score, p<0.05**.
